# Healthcare Access Experiences Among Indigenous Women in Northern Rural Thailand: A Focused Ethnographic Study

**DOI:** 10.5195/cajgh.2018.328

**Published:** 2018-11-06

**Authors:** Onouma Thummapol, Sylvia Barton, Tanya Park

**Affiliations:** 1 University of Alberta, Canada; 2 University of Northern British Columbia, Canada

**Keywords:** Indigenous women, Northern Rural Thailand, Ethnic minorities, Healthcare services, Access to healthcare, Gender equality, Human rights

## Abstract

**Introduction:**

Persistent inequities in health and access to healthcare services for indigenous women living in Thailand remain a significant challenge. This study provides narrative accounts of Indigenous women’s experiences accessing healthcare in northern and rural Thailand and explores the complexity of culture and its interaction with multiple intersecting influences on health behaviours.

**Methods:**

A focused ethnographic study was conducted to understand and describe the culture of health behaviors and other cultural phenomena. We recruited 21 female participants aged 20–41 years between March and April of 2017. In-depth semi-structured interviews conducted in Thai were used to explore the experiences of the participants living in a northern rural village. Data analysis was informed and guided by Roper and Shapira’s framework for ethnographic analysis.

**Results:**

Seven themes presented across three phases of experience (pre-access, making choices, and encountering difficulties) revealed an in-depth understanding of the Indigenous women’s lives, the broader sociocultural context in which they lived, and the challenges they faced when accessing healthcare. Analysis of data showed that the participants did not have equal access to healthcare and often disproportionately experienced discriminatory practices and negative attitudes of mainstream healthcare providers.

**Conclusions:**

This is the only study to date that discusses healthcare access challenges experienced by Indigenous women living in a northern rural Thai village. There is an urgent need to focus on citizenship, employment, and general health conditions; gender, familial, and labor roles; specific health conditions, wellness, and cultural practices; the seeking of healthcare services; healthcare provider relationships; the ability to access needed care; and optimization of self-care. Future efforts to improve healthcare access and reduce disease burden might benefit from these findings and allow for the development of more effective strategies, programs, and policies.

## Introduction

Access to healthcare is complex and multidimensional. Culturally diverse populations experience layers of complexity when accessing healthcare. Although access to quality healthcare is internationally recognized as a fundamental human right,^1^,^2^ inequities in health and healthcare access persist for many ethnic groups.^1^ Asian Indigenous women are more likely than non-Indigenous women to bear a greater burden of disease and have higher rates of suboptimal health.^2^ They are less likely to receive appropriate healthcare services and face a myriad of obstacles in their quest for achieving optimal health.^2^,^3^ In Asia, previously published studies provide evidence of the significant inequities for Indigenous and non-Indigenous populations. However, few studies have been published to understand healthcare access among Indigenous groups; thus Asian Indigenous health research remains largely overlooked.^2^,^4^–^5^ The existing literature shows that there are significant differences in Asian Indigenous women’s access and use of health services in comparison to the rest of the population.^3^,^6^–^8^ For example, Indigenous women are excluded from sexual and reproductive health services and are less likely to seek care when they are ill.^2^,^6^ The health status of Asian Indigenous women is a low priority and is often overlooked by the governments.^2^

Approximately 70% of the world’s Indigenous people reside in Asia.^2^ Thailand is a country in Southeast Asia, with 3,429 Indigenous villages and an Indigenous population of approximately 923,257 people.^2^,^4^ Indigenous peoples of Asia, including Thailand, as defined by the United Nations, are those who have a long connection with the lands and an experience of marginalization or discrimination, largely because they have a distinct and different cultural tradition and history compared to other groups within the state.^2^ While ‘Indigenous’ is a commonly-used international term, its use in Asia, particularly Thailand, is limited.^4^,^9^ In recognition of the right to self-identification declared in the United Nations Declaration on the Rights of Indigenous Peoples^2^, this paper primarily uses the term ‘Indigenous women’ to describe Indigenous women in Thailand.

In Thailand, many different Indigenous groups reside in northern rural highland areas. The government legally recognizes ten Indigenous groups, leaving almost half a million of Indigenous peoples in Thailand stateless.^4^ These stateless people can become subjects of discrimination and denial of basic human rights.^4^ Many of these stateless people are ethnic minorities living in northern rural areas near the Thai-Myanmar border.^10^–^11^

Health inequities exist for Indigenous groups in Thailand.^12^ Indigenous peoples often live in northern rural areas, where access to healthcare is limited or not available and these people are less likely to access public health services.^4^,^13^ This healthcare access inequity between Indigenous and non-Indigenous groups in Thailand can lead to negative health consequences. Many of the women who are affected by preventable diseases, such as cervical cancer^14^ and HIV/AIDS^15^, are Indigenous. They also experience multiple forms of discrimination and often have difficulty obtaining healthcare.^10^,^15^ This is attributed to the ‘double burden’ of Indigenous identity and gender inequalities.^12^

Globally, literature on Indigenous peoples’ healthcare access is available; however, few studies are gender specific.^2^ The current literature on healthcare access for Indigenous women is from countries such as Bangladesh^16^, Canada^17^–^19^, Guatemala^20^, India^7^–^8^, and Vietnam.^6^ These studies report on inequitable healthcare access and high disease burden; however, there is no Thai-specific literature. Furthermore, healthcare access experiences among Indigenous women in Thailand have not yet been explored. The purpose of this study is to understand the healthcare access experiences among Indigenous women in northern rural Thailand and to explore within these experiences the complexity of culture.

## Methods

Institutional Review Board human ethics approval of the study protocol was received in both Canada and Thailand. Informed written consent was obtained prior to conducting all of the interviews, which included a request to audio-record, as well as an opportunity to check back with participants to confirm the credibility of their responses. When obtaining informed consent, the researcher ensured that participants fully understood the purpose of the research, and potential risks/benefits associated with research participation. Participants were informed that they could make a voluntary decision to participate in the study, withdraw at any time, and choose not to answer any given question.

The research was a focused ethnographic study conducted using purposive sampling and snowball strategies guided by Knoblauch’s applied research methodology.^21^ Research questions included: 1) what are the experiences of Indigenous women accessing healthcare services in northern Thailand?; 2) how do cultural beliefs and practices influence access to healthcare for these women?; 3) what do Indigenous women believe are the facilitators and impediments to accessing quality healthcare?; and 4) how do these women experience engagement with healthcare providers in the healthcare system?

**Figure 1:**
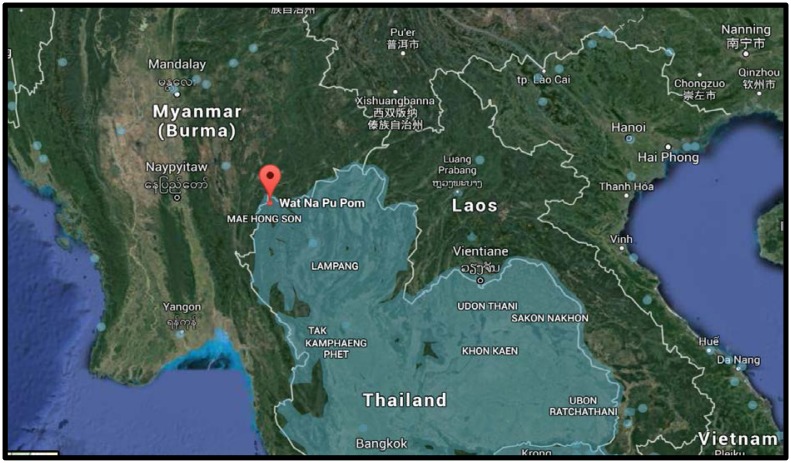
The study setting

The study site was a village located in the rural highland areas of Mae Hong Son province, one of the northern and mountainous provinces of Thailand that borders Myanmar (Figure 1). This province is approximately 924 kilometers (574 miles) away from the national capital, Bangkok. According to the Department of Provincial Affairs of Thailand (2014), ethnic groups represent approximately 63 % of the Mae Hong Son’s population. Participants were from the Tai-Yai group (also known as Shan); they have inhabited the rural highlands of Mae Hong Son for generations and make up the majority of the province’s populations.^22^ The village has a population of 457 Indigenous people and is located 55 kilometers (34.17 miles) from the closest hospital. It has poor road conditions and no access to public transportation.

### Data collection

Twenty-one women aged 20–41 years participated in interviews between March and April of 2017. We approached twenty-three women; however, due to language barriers and a lack of translation resources, two women who were not able to fully understand and communicate in Thai were excluded. Demographic data were collected from each participant at the beginning of the interview. The researcher identified and recruited participants using purposive and snowball sampling, the researcher’s personal networks, an Indigenous mentor (identified during a study feasibility trip), face-to-face approach, and study posters^23^–^27^. Data saturation dictated the sample size^28^–^30^ and occurred after 21 interviews. Field notes (e.g., observations and information recorded during the interviews) and a reflexive journal (e.g., the researcher’s thoughts, reactions to people and the setting, or personal feelings and emotions) were maintained during the course of fieldwork and used for analysis.^30^

The first author conducted face to face, in-depth interviews in Thai language with 21 women. These interviews focused on participants’ past and current experiences with the healthcare system. The interviews were semi-structured, with questions and probes that included: Where do you usually go for healthcare? Can you tell me about your last (or last two) healthcare visits? What influenced your decision to seek healthcare? How was it for you to get needed healthcare services? The interviews lasted approximately 45 minutes and were transcribed verbatim.

### Data analysis

Data collection and analysis proceeded concurrently after the first interview, with the first author transcribing and translating the first three interviews into English for review and analysis by the research team. The other transcripts were kept in the original language (Thai), with analysis completed by the first author. Roper and Shapira’s framework for ethnographic analysis guided the work using five strategies: a) coding for descriptive labels, (b) sorting for patterns, (c) identifying outliers or negative cases, (d) generalizing themes, and (e) noting reflective remarks (e.g., ideas or insights from the research team when collecting data and reviewing interview or relevant documents).^25^ NVivo qualitative data analysis software and a manual approach were used to organize and code narrative data.

Written materials compiled from field notes, a reflexive journal, and transcribed interviews were read and re-read prior to coding. Initial coding was discussed and refined by the research team and then grouped into meaningful, descriptive categories. These categories were then compared, contrasted, and sorted for patterns that reflected the similarities and differences between interviews, as well as for patterns related to cultural beliefs and practices. Following this pattern identification and notation, the data was re-read to abstract themes that captured the nature of the women’s experiences. Finally, this iterative process of data analysis resulted in organizing the seven themes across three phases of the women’s experiences, which was further discussed and refined by the research team, taking both emic and etic perspectives into the account.^25^

### Translation and Back-Translation

The interviews were conducted in Thai; however, English was the language used with the research team and reporting of the findings. Therefore, translation of the interviews was required. All audiotapes were transcribed verbatim in Thai. The first author read the transcribed transcripts in conjunction with the audiotapes to verify the accuracy of the transcription and to correct any transcription errors. The process of translation and back-translation began after themes and categories had been identified.^31^–^33^ Three people were involved in the translation and back-translation process: 1) the first author who conducted all of the interviews in Thai, 2) a Thai nurse researcher who conducted research in English and Thai, and 3) another Thai nurse researcher who conducted research in English and Thai.

The translated documents were compared and any discrepancies were resolved through discussion between the first two translators.^31^–^33^ The final English version was reached by agreement with the first two translators.^32^ To ensure the accuracy of translation, the process of back-translation was conducted by the third translator.^31^–^33^ To achieve equivalence between original and targeted languages, and to avoid misrepresentation of participants’ feedback, the first researcher compared the back-translated transcript with the original version.^31^–^33^ The translation and back-translation processes were repeated multiple times and discussed throughout, in order to minimize any discrepancies in meaning.^34^

It has been well recognized that achieving equivalence between two languages is a challenge.^35^ Therefore, working with a bilingual translator who possessed an understanding and knowledge of the participants’ culture and language, and was familiar with medical terminology and research, was an optimal way to produce accurate and meaningful data.^32^–^33^, ^35^

## Results

Three phases: pre-access, making choices, and encountering difficulties which integrate seven overlapping themes exemplify participants’ experiences accessing healthcare. Table 1 presents the demographic characteristics of the participants and Table 2 lists the themes and their definitions.

### Pre-access

The pre-access phase of healthcare access focused on the participants’ experiences of employment and income, gender roles and responsibilities, and views on health and treatment.

The majority of participants were self-employed (farmers, shop owners, and cooks), while others were government-employed schoolteachers. The self-employed participants reported that they faced additional difficulties, such as fear of income loss when taking time away from work for medical visits. One participant stated, “I was crying in pain at work because I could not afford to take my day off to go to see the doctor…The employer will only pay me 100–200 Baht [$ 3–6 USD] a day and if I take a day off work we will not have money to buy food…” (Participant 11). In addition to unpaid work in the household, participants also engaged in paid work to supplement family income and explained that they had little time to think about their own health needs, let alone access healthcare. One participant shared, “Besides cooking, doing household chores and looking after children and the family, I’m also working on the farm…I have to do everything and have no time for myself” (Participant 4).

Participants reported that they usually met gender expectations related to their domestic and reproductive roles. One participant stated, “…cooking, washing clothes, and caring for children are our responsibility…we work within and outside the home. So caring for children and going to the hospital…it’s hard for me” (Participant 3). Participants also mentioned having to assume unpaid caregiving duties for the ill, elderly, and young children had significant impact on their decisions to seek healthcare. One participant explained, “I have two siblings but they are not here. I live with my mom and have to look after her and take care of everything…like going to town to fill her prescriptions…I will not go to see the doctor unless I’m really sick because I don’t want to leave my mom alone” (Participant 2).

During many of the interviews, traditional beliefs and practices during the postpartum period were described, such as herbal steam baths, keeping the body covered from head to toe, and food beliefs. One participant stated, “Herbal steam baths made by boiling a mixture of herbs such as roots and leaves will promote a mother’s perspiration which eliminate residual impurities, improve the skin and stimulate breastmilk” (Participant 6). Another participant shared, “Keeping the body of both, mother and baby covered from head to toe for at least a month after delivery is important to prevent further cooling. We believe it will give strength and protect mother and baby from getting sick” (Participant 5).

### Making choices

The making choices phase of healthcare access focused on the participants’ experiences of traditional and Western medicines, support networks and resources, the referral system, and previous experiences.

All participants used a combination of traditional and Western practices, which were largely influenced by the perceived nature and severity of health conditions. As one participant explained, “If you were not feeling well and did not get better after visiting the doctor, you have ‘lom nok’ [physical symptoms for which the doctor can find no cause, which was believed to be caused by supernatural forces]. I would go to see a traditional healer for religious rituals and drink ‘nam mon’ [holy water] and then I would get better” (Participant 7). Several participants mentioned health conditions that they believed to be caused by demons or supernatural forces. One participant revealed, “When my son was three months old, he cried inconsolably for several nights for no apparent reason. I took him to the village traditional healer at night for healing, who got him to drink ‘nam mon’ [holy water] and put ‘sai sin’ [a white holy cord] around his neck. My son got better” (Participant 17).

Positive social support and resources from family, neighbors, and community were mentioned as important factors contributing to participants’ decisions about health and access to healthcare services. Several narrative accounts emerged regarding support systems. For example, during a crisis, neighbors offered rides to the doctor. One participant stated, “I did not make it to the hospital to deliver my first child. The village vehicle at the community clinic was not available, but luckily one of our neighbors drove me to the district hospital. The health worker at the community clinic tagged along and I gave birth to my child in the car on our way to hospital.” (Participant 12).

All participants discussed healthcare services that they accessed inside or outside the village as a referral system for sub-district (traditional healer, traditional herbs or medicines, and community health center), district (private clinics, drug stores, and district hospital), and hospitals within and outside the provincial areas. The majority of the participants primarily accessed the community health centers when experiencing symptoms of illness, while some participants bypassed the community health centers and directly accessed the hospitals, often paying extra for this. The latter group was more educated, had a higher income, had experience with medical conditions, and/or knew someone working in healthcare setting. One participant shared, “I will go to the [provincial] hospital when I’m sick because I like the quality of services there better, even though I have to pay extra for it. I paid 800 baht [equivalent to $23 USD] for blood tests because it was not covered by universal healthcare when you bypassed the community health center and district hospital” (Participant 19).

Throughout the interviews, it became apparent that participants’ opinions of the quality of the healthcare they have received was highly dependent on the interactions with their healthcare providers. Participants reported feeling that they were respected, accepted, and cared for, particularly when a strong positive relationship/interaction was formed. The presence of Indigenous health providers, in particular, was perceived as crucial because of the shared cultural and linguistic heritage, which created an environment of belonging and acceptance. As one participant stated, “I really appreciated that I got to see an Indigenous nurse when I visited the district hospital. She was very friendly and understanding...made me feel at ease” (Participant 3).

### Encountering difficulties

The encountering difficulties phase of healthcare access focused on the participants’ personal characteristics and circumstances, transportation and distance, as well as racism and discrimination.

The majority of participants reported having Thai citizenship. Participants who had citizenship status generally reported that they were able to access basic education, healthcare and social services, and had freedom of movement and employment opportunities. Participants who did not have Thai citizenship reported difficulties in achieving the rights and benefits given to ‘citizens’. The following excerpt illustrates challenges undocumented participants faced when attempting to obtain essential care, “I took my child to the community clinic early in the morning on a weekend because of fever and got yelled at by the health worker… she was not friendly and refused to provide care, saying, “Come back during the operating hours.” I did not want to go there again and would rather treat my son’s symptoms myself” (Participant 17). Another undocumented participant stated, “I do not have citizenship… I am stuck here, I cannot go anywhere I want to” (Participant 16).

Multiple intersecting influences affecting participants’ access to healthcare were identified throughout the interviews. These included specific transportation and distance challenges due to the rural and mountainous geography, contending with time away from work, long wait times, financial constraints, family responsibilities, severity of symptoms, and cultural discrimination. One participant shared, “I could spend a day waiting to see a doctor. It’s a waste of time. I would rather not go and suffer, wait for the symptoms to go away on their own. It’s difficult, we are living far from town and if we are not extremely sick, we won’t go. Many of us are poor and do not have a vehicle, asking others who have private vehicles to drive us, which cost thousands of Baht, not to mention cost of food. Transportation alone will cost 1000 Baht [$29 USD]” (Participant 5). Several suggestions were made regarding how to improve access to healthcare services, and included reducing wait times, making the waiting room more inviting and welcoming, providing outreach services in the village, and establishing a transport system, especially in case of emergency. One participant shared, “I would like them [health care providers] to come to the village…every once in a while if possible, for services like pap smears or physical check-ups for those who are ill. We are poor and many do not have money to go to the hospital, so they would miss the appointment. If they [healthcare providers] brought the services to us, it would save us a lot” (Participant 7).

Many participants reported a lack of trust in non-Indigenous healthcare providers resulting from firsthand experience of discriminatory and insensitive behaviors; such as unfriendliness, lack of attention, and negative reactions to Indigenous accent, appearance and a lack of education. One participant shared, “The last time I was there [district hospital] was when I had an ankle sprain from a motorcycle accident. I was not okay with the way I was treated. I felt like the doctor did not respect me as a patient when he talked to me. I wished he knew how to treat patients fairly and not based on how we look or how much money we have” (Participant 5).

## Discussion

The findings of this study highlight the significance of personal, socioeconomic, cultural/geographical contexts, and previous healthcare experiences that affect participants’ access/use of healthcare services. These experiences provide a window into understanding the complexity of culture affecting healthcare access inequitties.

One striking aspect of the findings is related to gender inequality. The participants’ accounts of life based in traditional and patriarchal norms perpetuate their status as subordinate, creating a ‘double burden’ for women.^36^ The unequal distribution of responsibilities (within and outside of the home) leads to unequal opportunities to seek care, invest in educational and vocational skills, and participate in paid work.^36^–^40^ These unequal opportunities offer an important snapshot into how cultural values and gender roles affect health inequities. The United Nations Sustainable Development Goal 5 (SDGs) provides strong support for the action to reduce health inequities for Indigenous women.^41^ Action priorities include the recognition of women’s roles, implementation of strategies to change cultural and social norms that can form barrier to equal opportunity, and better integration of gender perspectives into the healthcare system.^41^–^42^ This will, in turn, ensure effective use of healthcare services and reduce health inequities.

Understanding women’s unique health needs and experiences, the differences in how they view and take care of their health, their cultural and religious beliefs and practices related to health and decisions-making, are all critical in identifying appropriate strategies and interventions to enhance healthcare access. For example, the findings revealed that participants are more likely to self-treat or use alternatives (e.g., traditional medicines), depending on the perceived cause and severity of conditions. Domestic duties prevent women from going to the hospital during operating hours, resulting in adverse consequences on their health. In addition, the findings showed that participants were not aware of the benefits and importance of preventive health services. This suggests the necessity of providing linguistically and culturally appropriate education, in order to increase women’s participation in screening and early detection.^14^,^18^,^20^ The results also demonstrated that women adhered to beliefs and taboos on food during the postpartum period. These findings parallel the extensive literature suggesting the importance of integrating holistic approaches to health into mainstream healthcare practices and policies, such as the development of culturally appropriate nutritional programs.^43^ Programs that could improve pregnancy outcomes and long-term quality of life for this underserved population are needed to be implemented.^43^

Participants are subject to complex and different socioeconomic and cultural influences that impact their options for healthcare practices. Our findings indicated that healthcare services were not available to the participants on an equitable basis due to geographic factors. This introduces financial costs associated with access to childcare, transportation, accommodation, and etc., which leads to further health inequity for women. The literature that has explored this issue among Indigenous women supports this view.^7^,^18^,^44^ Although the use of traditional and Western medicines is commonplace, the findings raise concerns about equitable healthcare access and highlight factors associated with avoidance or delayed access. Understanding why women fail to seek or come late for care can inform efforts and interventions to reduce treatment delay; which will result in early detection of disease, timely care, increased survival, and ultimately, desired health outcomes.^7^,^45^–^46^

This study revealed that structural factors, including long wait times and the discriminatory behavior of healthcare providers, negatively affect participants’ experiences, satisfaction, and decision to engage with healthcare. Participants described how experiences of unequal treatment, discrimination, and long waits discouraged them from seeking healthcare and developing trusting relationships with care providers. These findings corroborate several studies suggesting that strong, respectful and trusting relationships, as well as meaningful and familiar connections, are integral for encouraging care seeking.^47^–^48^ It also highlights the need for education and professional development to increase the sensitivity, compassion, and reflective practices of healthcare providers.^3^,^49^–^50^

The three access phases and seven integrated themes revealed the intersectionality of influences on the participants’ health and access to healthcare. Many participants faced additional challenges that interfered with the enjoyment of their human rights, particularly in relation to the right to healthcare on the basis of factors such as ethnicity, gender roles, cultural beliefs and practices, socioeconomic status, and geography.^51^–^52^ Participants were further affected by discrimination as Indigenous people, and therefore access to healthcare was further limited. This study offers insight and understanding into Indigenous women’s lives, the broader sociocultural context in which they live, and the challenges they face when accessing healthcare. Intersectionality is particularly useful in informing such analyses because it draws attention to multiple intersecting social influences and how they shape the ways in which healthcare is experienced, received, and provided.^51^^–53^ Understanding the intersections and taking into account women’s perspectives will enable policy makers and health care providers to better design context-specific strategies that will improve equitable access to healthcare.

There are several limitations to this project. The first author interviewed a small and select sample of Thai speaking participants who had similar backgrounds and social levels (educational, geographical, or occupational). In particular, the research setting was relatively homogeneous with respect to gender and ethnicity. As is essential in qualitative research, the participants were selected based on their ability to provide information in Thai, not on the basis of how they represented the general population. It is acknowledged that the qualitative research results are not generalizable, and that the participants may not be representative of the general population. Given the lack of translation facilities, participants who did not speak Thai were excluded; this may have resulted in the study not capturing the healthcare challenges of non-Thai-speaking Indigenous participants who may have different experiences.

Future research is needed on healthcare access inequalities for Indigenous women in Thailand. It can replicate the findings of this study within similar contexts, such as northern and rural communities across Thailand, supporting the urgent need to improve healthcare access for Indigenous women. Another area for future research would be on exploring the experiences of healthcare providers working with Indigenous communities, which may enhance the understanding of gaps and inform strategies to remove or minimize barriers to access.

In this study, we identified the impacts of gender and family roles on the cultural understandings and experiences in seeking healthcare among Indigenous women. It would be interesting to examine in-depth the impact of Indigenous women’s illnesses or diseases on family function and relationships. Thus, ethnographic studies with this focus are recommended. Last, given the lack of disaggregated data on Indigenous peoples in Thailand and of culturally relevant indicators, research is warranted pertaining to Indigenous data collection on gender, race/ethnicity, culture, Indigenous identity, health status, socioeconomic status, or geography. Collecting and using such data is important to advance human rights, achieve equal opportunities, redress discriminatory disadvantages, and improve Indigenous health outcomes.

This study might potentially have important implications for current gender policy discussions. Gender equality and women’s rights are increasingly recognized by international institutions, such as the United Nations Sustainable Development Goal and the Convention on the Elimination of Discrimination Against Women (CEDAW). Because of disadvantaged position in society, women who belong to minority groups suffer disproportionately from multiple forms of discrimination and are less likely to enjoy the full enjoyment of human rights, including equitable access to quality healthcare. Thus, the results of this study can assist advocates, policy makers, and allied healthcare professionals to understand the differences among women with respect to multiple influences and contexts (e.g., socioeconomic, cultural, ethnic, citizenship, and geographical).

This is the only study to date that discusses healthcare access challenges experienced by Indigenous women living in a northern rural Thai village; emphasizing an urgent need to enhance non-discriminatory access to, and quality delivery of, healthcare services to Indigenous women in northern rural Thailand. Future research should focus on equitable healthcare access and disease burden reduction, in order to develop effective strategies, culturally sensitive programs, and evidenced-based policies.

## Figures and Tables

**Table 1:** Demographic Characteristics of the Study Sample of 21 Participants

Characteristics	N	%
Age (mean = 34)		
≤20	2	10
21–30	7	33
31–40	5	24
≥41	7	33
Place of birth		
Home birth	18	86
Institutional birth	3	14
Citizenship status		
Thai citizenship	18	86
Non-citizenship status	3	14
Highest education level		
Some high school, no diploma	9	43
High school graduate	7	33
Some college credit	2	10
Bachelor’s degree	2	10
Master’s degree	1	4
Occupation		
Employed	20	96
Farmer	14	68
School teacher	3	14
Small shop owner	2	10
Cook at school	1	4
Unemployed (student)	1	4
Monthly Income (in Baht [34.40 Baht = 1 USD, Rates on April 30, 2017])		
<5000	10	48
5000–10000	6	29
11000–15000	3	14
16000–20000	1	4
21000–25000	1	4
Health conditions		
Chronic (at least one)	9	43
Non-chronic	20	95

Chronic health conditions included high blood pressure, high cholesterol, diabetes, thyroid dysfunction, and ear, nose, and throat problems.

The non-chronic health conditions included accidents/injuries, dental issues, tropical disease (malaria), skin problems, drug allergies, headache, and anxiety.

**Table 2:** List of Themes and Their Definitions

Understanding the lives of the women	Non-citizen women have restricted access to care Health-seeking behaviour is influenced by employment
Women’s roles in Thai society	More disadvantaged and vulnerable to poor health outcomes The expected gender roles create ‘double burden’
Wellness and cultural practices	Caregiving as a female responsibility Unequal distribution of responsibilities Reproduction, chronic conditions, and accidents are most common issues Traditional herbs/medicines, farm work, and religious rituals used to stay healthy Some cultural specific practices and religious beliefs influence health and decisions about care
Seeking out healthcare services	The provision of services not equally distributed Traditional and Western medicines are complementary Structural barriers and socioeconomic characteristics identified as reasons for avoidance or delayed access
Engaging with health care providers	Having familial connections is described as beneficial
Navigating access to quality healthcare	A sense of belonging and acceptance created by meaningful interactions A discriminatory and insensitive behaviours A desire to engage enhanced when feeling treated with respect
Facilitating healthcare access	Unequal treatment and discrimination based on different characteristics Long waiting time viewed as the most challenging The complexity of gender and its intersection between social categories Strong family and community relationships promoted care seeking and outcomes Need for reducing wait times; better healthcare environment; outreach services; and transport system

## References

[1] World Health Organization (2015). Health and Human Right [Internet].

[2] United Nations (2015). State of the world’s Indigenous peoples (2nd volume): Indigenous peoples access to health services [Internet].

[3] Thummapol O, Park T, Barton S (2018). Exploring health services accessibility by Indigenous women in Asia and identifying actions to improve it: A scoping review. Ethnicity and Health.

[4] Dhir RK (2015). Indigenous Peoples in the world of work in Asia and the Pacific: A status report [Internet].

[5] United Nations (2010). State of the world’s Indigenous peoples (Volume 1) [Internet].

[6] Dang HA, Gillette H, Harry P (2012). Vietnam: A widening poverty gap for ethnic minorities [Internet]. Indigenous Peoples, Poverty and Development.

[7] Jose JA, Sarkar S, Kumar S, Kar SS (2014). Utilization of maternal health-care services by Tribal women in Kerala. Journal of Natural Science, Biology & Medicine.

[8] Shah R, Bélange D (2011). Socioeconomic correlates of utilization of maternal health services by Tribal women in India. Canadian Studies in Population.

[9] Asia Indigenous Peoples Pact (2014). Overview of the state of Indigenous peoples in Asia [Internet].

[10] Cadchumsang J (2011). People at the rim: A study of Thai ethnicity and nationalism in a Thai border village [dissertation].

[11] Spindler W (2015). Thousands of stateless people given nationality in Thailand [Internet].

[12] Lutvey T (2014). Gender mainstreaming manual: Good practices and lessons learnt of an Indigenous Peoples organization [Internet].

[13] United Nations (2010). High Commissioner for Refugees. Good practices addressing statelessness in South East Asia [Internet].

[14] Kritpetcharat O, Sirijaichingkul S, Kritpetcharat P, Wutichouy W (2012). Comparison of pap smear screening results between Akha hill tribe and urban women in Chiang Rai province, Thailand. Asian Pacific Journal of Cancer Prevention.

[15] Apidechkul T (2016). A 20-year retrospective cohort study of TB infection among the hill-tribe HIV/AIDS populations, Thailand. BMC Infectious Diseases.

[16] Chowdhury HE (2017). Framework for tribal peoples plan-health sector support program [Internet].

[17] Denison J, Varcoe C, Browne A (2014). Aboriginal women’s experiences of accessing health care when state apprehension of children is being threatened. Journal of Advanced Nursing.

[18] Morgan L, Wabie J (2012). Aboriginal women’s access and acceptance of reproductive health care. A Journal of Aboriginal & Indigenous Community Health.

[19] Van Herk K, Smith D, Andrew C (2011). Identity matters: Aboriginal mothers’ experiences of accessing health care. Contemporary Nurse.

[20] Schooley J, Mundt C, Wagner P, Fullerton J, O’Donnell M (2009). Factors influencing health care-seeking behaviours among Mayan women in Guatemala. Midwifery.

[21] Knoblauch H (2005). Focused ethnography. Forum Qualitative Social Research.

[22] Ord M (2015). The Travelfish [Intternet].

[23] Bonevski B1, Randell M, Paul C, Chapman K, Twyman L, Bryant J, Brozek I, Hughes C (2014). Reaching the hard-to-reach: A systematic review of strategies for improving health and medical research with socially disadvantaged groups. BMC Med Res Methodol.

[24] Liamputtong P (2007). Researching the vulnerable.

[25] Roper J, Shapira J (2000). Ethnography in nursing research [Electronic resource].

[26] Sixsmith J, Boneham M, Goldring J (2003). Accessing the community: Gaining insider perspectives from the outside. Qualitative Health Research.

[27] Higginbottom G, Pillay J, Boadu N (2013). Guidance on performing focused ethnographies with an emphasis on healthcare research. Qualitative Report.

[28] Cruz EV, Higginbottom G (2013). The use of focused ethnography in nursing research. Nurse Researcher.

[29] Guest G, Bunce A, Johnson L (2006). How many interviews are enough? An experiment with data saturation and variability. Field Methods.

[30] Ortlipp M (2008). Keeping and using reflective journals in the qualitative research process. Qualitative Report.

[31] Al-Amer R, Ramjan L, Glew P, Darwish M, Salamonson Y (2015). Translation of interviews from a source language to a target language: Examining issues in cross-cultural health care research. Journal of Clinical Nursing.

[32] Chen H, Boore J (2010). Translation and back-translation in qualitative nursing research: Methodological review. Journal of Clinical Nursing.

[33] Nurjannah I, Mills J, Park T, Usher K (2014). Conducting a grounded theory study in a language other than English. Sage Open.

[34] Maneesriwongul W, Dixon J (2004). Instrument translation process: A methods review. Journal of Advanced Nursing.

[35] Squires A (2008). Language barriers and qualitative nursing research: methodological considerations. International Nursing Review.

[36] Ferrant G, Pesando GL, Nowacka K (2014). Unpaid care work: The missing link in the analysis of gender gaps in labour outcomes.

[37] Binder-Finnema P, Lien P, Hoa D, Målqvist M (2015). Determinants of marginalization and inequitable maternal health care in North-Central Vietnam: A framework analysis. Global Health Action.

[38] Munro J, McIntyre L (2016). (Not) getting political: Indigenous women and preventing mother-to-child transmission of HIV in West Papua. Culture, Health & Sexuality.

[39] Norsa’adah B, Wnorlida W (2014). Preventive health practices among women at rural villages in Malaysia. International Medical Journal.

[40] Wongwatcharanukul L, Promthet S, Bradshaw P, Jirapornkul C, Tungsrithong N (2014). Factors affecting cervical cancer screening uptake by Hmong hilltribe women in Thailand. Asian Pacific Journal of Cancer Prevention.

[41] United Nations (2015). Sustainable Development Goals. Sustainable development goal 5: Achieve gender equality and empower all women and girls [Internet].

[42] Sen G, Ostlin P (2007). Unequal, unfair, ineffective and inefficient gender inequity in health: Why it exists and how we can change it [Internet].

[43] Riang’a R, Nangulu A, Broerse J (2017). Food beliefs and practices among the Kalenjin pregnant women in rural Uasin Gishu County, Kenya. Journal of Ethnobiology and Ethnomedicine.

[44] Browne A (2010). Issues affecting access to health services in northern, rural and remote regions of Canada.

[45] Lama S, Krishna A (2014). Barriers in utilization of maternal health care services: Perceptions of rural women in Eastern Nepal. Kathmandu University Medical Journal.

[46] Cameron B, Carmargo Plazas M, Salas A, Bourque Bearskin R, Hungler K (2014). Understanding inequalities in access to health care services for aboriginal people: A call for nursing action. Advances in Nursing Science.

[47] Askew D, Brady J, Brown A, Cass A, Davy C, DeVries J, Fewquandie B, Hackett M, Howard M, Ingram S, Liu H, Mentha R, Peiris D, Simon P, Rickards B, Togni S (2008). To your door: Factors that influence Aboriginal and Torres Strait Islanders peoples seeking care. Kanyini Qualitative Study Monograph Series.

[48] Davy C, Cass A, Brady J, DeVries J, Fewquandie B, Ingram S, … Brown A (2016). Facilitating engagement through strong relationships between primary healthcare and Aboriginal and Torres Strait Islander peoples. Australian & New Zealand Journal Of Public Health.

[49] Castro A, Savage V, Kaufman H (2015). Assessing equitable care for Indigenous and Afrodescendant women in Latin America. Revista Panamericana De Salud Publica-Pan American Journal of Public Health.

[50] Sharma B, Ramani K, Christensson K, Giri G, Johansson E (2013). The transition of childbirth practices among tribal women in Gujarat, India - a grounded theory approach. BMC International Health and Human Rights.

[51] Hankivsky O, Reid C, Cormier R, Varcoe C, Clark N, Brotman S (2010). Exploring the promises of intersectionality for advancing women’s health research. International Journal for Equity in Health.

[52] Iyer A, Sen G, Östlin P (2008). The intersections of gender and class in health status and health care. Global Public Health.

